# Prior CT imaging history for patients who undergo whole-body CT for acute traumatic injury and are discharged home from the emergency department

**DOI:** 10.1186/s12873-018-0186-1

**Published:** 2018-10-16

**Authors:** Mary Matthews, Peter Richman, Scott Krall, Kimberly Leeson, K Tom Xu, Albert L Gest, Osbert Blow

**Affiliations:** 1 Department of Emergency Medicine, CHRISTUS HEALTH/Texas A&M Residency in Emergency Medicine, Corpus Christi, TX 78404 USA; 2 Department of Emergency Medicine Texas Tech College of Medicine, Lubbock, TX USA; 3 Department of Acute Care Surgery, Trauma & Surgical Critical Care, CHRISTUS Spohn Hospital, Corpus Christi, TX USA

**Keywords:** Acute traumatic injury, Discharge from ED, CT scans

## Abstract

**Background:**

Recurrent CT imaging is believed to significantly increase lifetime malignancy risk. We previously reported that high acuity, admitted trauma patients who received a whole-body CT in the emergency department (ED) had a history of prior CT imaging in 14% of cases. The primary objective of this study was to determine the CT imaging history for trauma patients who received a whole-body CT but were ultimately deemed safe for discharge directly home from the ED.

**Methods:**

This was a retrospective cohort study conducted at an academic ED. All trauma patients who were discharged directly home from the ED after whole-body CT were analyzed. The decision to utilize whole-body CT was at the discretion of the caring physician during the study period. Clinical data for the most recent trauma visit was recorded in a structured fashion on a standardized data collection instrument utilizing the hospital system electronic medical record (EMR). Subsequently, study investigators reviewed a shared, electronic radiological archive for the 6-hospital system to evaluate prior CT exposure for each patient.

**Results:**

165 patients were in the study group. The mean age of the study group was 39+/− 16 years old, 40% were female and 64% were Hispanic. The most common mechanism of injury in our study group was motor vehicle crash (MVC) (66%). In our study group, 25% had at least one prior CT. The most common prior studies performed were: CT abdomen/pelvis (13%), CT head (9.1%), CT face (6.7%), and CT chest (1.8%). Within a multivariate logistic regression model we found that the large majority of patient characteristics and mechanisms of injury were not associated with a positive prior CT imaging history.

**Conclusion:**

We found a positive history for prior CT for 25% of trauma patients who received whole-body CT scan but were discharged from the ED to home.

## Background

CT imaging has the capacity to detect a variety of different pathologies encountered in the emergency department (ED). Rosen described that CT is beneficial to the emergency medicine physician by increasing the certainty of diagnosis within a short period of time and significantly decreasing the need for surgical exploration [1]. In light of these benefits, CT use in the ED increased from 2 million studies (1980) to 72 million (2007) [2]. More recently, Levin reported that CT use in the ED tripled from 2002 to 2012, rising from 57.2 scans to 147.9 scans per 1,000 patient encounters [3].

One area, in particular, for which there has been significant expansion in CT utilization is the evaluation of trauma patients. From 1998 to 2007 there was a national 3-fold increase in the use of CT scans in the ED for injury-related conditions [4]. Such an increase is undoubtedly due to the potential for such imaging to reduce the risk of missed injury. CT scanning has been shown to increase diagnostic accuracy for blunt trauma patients compared to conventional radiological imaging. Several investigators have reported that whole-body (brain, c-spine, trunk) CT frequently identifies unexpected findings and can lead to a change of treatment as high as 33% of patients [5–7].

When considering the increased use of CT imaging, clinicians must weigh the risks inherent to this modality. A single whole-body CT provides significantly more radiation exposure than a conventional x-ray, and at a dose in excess of which is felt to be safe within a one-year period, by the International Symposium on the System of Radiological Protection. (20 mSv) [8]. Reviews of published analyses suggest whole-body CTs could directly result in cancers as often as 1 in 380 and cause 12.5 additional cancer deaths in 10,000 patients [9]. Furthermore, CT scans in children will result in significantly increased lifetime radiation risk over adult CT, both because of the increased dose per milliampere-second, and the increased lifetime risk per unit dose [10]. With increased CT use, it is prudent to assess whether patients understand the risks associated with this modality. Investigators recently reported that patients were more confident when CT imaging was part of their medical evaluation, but, unfortunately, they had a poor understanding of the concomitant radiation exposure/risk and underestimated their previous imaging experience [11].

A recent study published at our center in 2015 revealed that 14% of trauma patients that received whole-body CT and were admitted to the hospital had prior CT imaging before their traumatic injury [12]. This study was limited in that it focused on a patient population that represents the subset of trauma patients with more significant injuries such that whole-body CT was likely unavoidable. On the other hand, there is a segment of trauma patients with significant mechanisms for which there may be some risk of injury but clinical suspicion may be more moderate or even low. These patients represent an opportunity for shared decision-making where clinicians may discuss the balance between clinical concerns for serious injury vs. the risk of imaging. We believe physicians will find it helpful in that context to understand the prior imaging exposure for this cohort of trauma patients. For that purpose, we conducted a retrospective cohort study to examine the imaging history for trauma patients who underwent a whole-body CT but were ultimately considered safe from discharge from the ED.

## Methods

### Study design

This was a multicenter, retrospective cohort study to assess the prior imaging history for patients with significant trauma mechanisms and clinical suspicion for important traumatic injuries that were ultimately deemed to be safe for discharge from the ED to home following whole-body CT. Data from all trauma patients who were discharged directly home from the ED after whole-body CT from 3/25/2016 to 10/25/2016 were analyzed.

### Setting

The study was conducted at Christus Spohn Memorial Hospital (site of patient enrollment) and electronic records were reviewed from Memorial Hospital along with five other Christus Spohn hospital sites. Christus Spohn Memorial is a major teaching affiliate of Texas A&M College of Medicine, a level-two trauma center, and serves an inner city population. The annual ED census is 45,000 patients. The six affiliated hospitals comprise 192,073 annual ED visits, which, based on administrative data available locally, constitutes approximately 70% of all ED visits within our twelve-county region of southern Texas. The study was approved by the Spohn Institutional Review Board prior to initiation of data collection.

### Population

The study included all adult ED patients, age > 17 years, from 3/25/16–10/25/16 for whom there was clinical suspicion for important traumatic injuries but were ultimately deemed by the emergency physician to be safe for discharge home following whole-body CT (i.e. patients discharged home from the ED). Patients were excluded if they were pregnant, prisoners, admitted to the hospital from the ED, or had less than a full whole-body CT to evaluate their traumatic injuries. Inclusive electronic radiological logs were reviewed on a weekly basis by study authors to include any patients that were not identified at point of care.

### Study protocol

Evaluation of trauma patients and the decision to utilize whole-body CT was at the discretion of the caring physician during the study period. Clinical data for the current visit were recorded in a structured fashion on a standardized data collection instrument utilizing data from the hospital system EMR with all study data points determined a priori. In a similar structured fashion, study investigators reviewed a shared, electronic radiological archive for the 6-hospital system to evaluate prior CT exposure for each patient.

### Statistical analysis

Data were entered into Excel 2013 for Windows (Microsoft Corporation, Redmund, WA), and, then, transported into STATA 11 software (StataCorp LLC, College Station, TX) for statistical analysis. Continuous data are presented as means +/− standard deviations and were analyzed by t-tests. Categorical data are presented as frequency of occurrence and were analyzed by chi-square. We calculated odds ratios with 95% CIs.

Our primary outcome parameter was the percentage of patients who were discharged from the hospital from the ED following whole-body CT and determined to have had a prior history of CT(s) before their traumatic event. Prior CT was defined as CT imaging of any protocol/body area identified on a dedicated page within our system-wide EMR. Data was available for review from this system from 2009 to the present time.

As bivariate analyses often mask confounding of other independent variables, we utilized multivariate logistic regression to delineate the effect of each independent variable. The dependent variable was whether patients had any CT(s) performed previously, The independent variables were sex, age, race/ethnicity and mechanism of injury. In view of our methodology for data collection that involved chart review, we were limited to analyzing patient characteristic variables available in the EMR. Physician and examination characteristics were not included within the model.

## Results

Over the seven-month period, 165 patients were discharged home from the ED after receiving whole-body CT scan. The mean age of the study group was 39 (SD+/− 16) years old, 40% were female (66/165), and 64% (106/165) were Hispanic. The most common mechanism of injury in our study group was MVC (66%; 109/165). Other mechanisms included fall (6.7%; 11/165), stab head (11%; 18/165), and other (16%; 27/165).

Within the study group, one-fourth (25%; 41/165, 95% CI = 19–32%) had at least one prior CT. The prior studies performed in order of frequency were: CT abdomen/pelvis 13% (21/165), CT head 9.1% (15/165), CT face 6.7% (11/165), CT cervical spine 5.5% (9/165), CT limb 4.2% (7/165), CT thoracic spine 3%(5/165), CT lumbar spine 3%(5/165), CT neck 1.8% (3/165), and CT chest 1.8% (3/165).

With respect to aggregate exposure to chest and/or abdominal CT, studies with typically higher doses of radiation, we found 11 patients (6.7%) had at least one previous study prior to enrollment in our study, five (3%) had two previous studies, and one patient (0.6%) has 13 previous CT studies.

A multivariate model which included patient characteristics and mechanism injury found that age 41–64 years (*p* = .025, OR = 2.6; 95% CI 1.12–6.14) and “other” trauma mechanisms (non-fall, non-stab wound to head, non-MVC; (*p* < 0.001; OR 7.40; 95% CI 2.6–21) were associated with positive prior CT history We did not find that other patient characteristics were associated with a prior CT history, including age > 64 years (*p* = 0.098; OR; 95% CI 0.78–20) female gender (*p* = 0.84; OR 0.92; 95% CI 0.39–2.1), Hispanic race (*p* = 0.82; OR 0.90; 95% CI 0.37–2.2), non-white/non-Hispanic race (*p* = 0.73; OR 0.73; 95% CI 0.14–3.78), fall mechanism (*p* = 0.86; OR 1.2; 95% CI 0.22–6.1), and stab wound head (*p* = 0.068; OR3.2; 0.92–11).

A similar multivariate model found that age 41–64 years (*p* = .03, OR = 1.0; 95% CI 1.0–1.1) was associated with positive prior CT abdomen and/or chest history. We did not find that other patient characteristics were associated with such an imaging history, including age > 64 years (*p* = 0.054; OR 6.1; 95% CI 0.96–39), female gender (*p* = 0.88; OR 0.92; 95% CI 0.32–2.6), Hispanic race (*p* = 0.39; OR 1.7; 95% CI 0.50–6), non-white/non-Hispanic race (*p* = 0.38; OR 2.4; 95% CI 0.33–18), stab wound head (*p* = 0.15; OR 2.8; 95% CI 0.43–6.5), and other non-MVC, no stab wound head mechanisms (*p* = 0.46;; OR 1.7; 95% CI 0.43–6.5).

## Discussion

We found that 25% of patients within our cohort who received a whole-body CT but then were deemed low risk for discharge had a prior history of CT imaging. Of note, 6% of the study group had more than one prior CT in our health system. We believe our results provide a context for clinicians to understand the risk of prior imaging beyond their reliance on patient recall and single hospital records. As much as physicians might desire a complete imaging history, for a variety of reasons the available information may be limited. Many patients have had CTs in unrelated hospitals and private imaging centers. Furthermore, the accuracy of patient recall is poorly understood to date. While we cannot account for imaging performed in private centers and in distant locations, our 6-hospital system provides a significant proportion of the care for patients within a 12-county area, thus, providing a better window to evaluate previous exposure for our patients compared with most centers.

Our study fits within the context of the growing body of literature on CT utilization in the setting of trauma and efforts to limit radiation exposure to those most likely to have medically important injuries. In recent years, a significant proportion of patients who come to the ED after a high-energy traumatic event undergo whole-body CT Scan for evaluation of suspected injuries [11]. In view of the associated radiation risks, investigators have worked to identify patient characteristics that reduce the likelihood that CT will identify unexpected injuries [13–15]. Linder et al. conducted a two-center retrospective cohort study of 523 alert trauma patients for which 139 were classified as low risk [15]. The authors did not identify any significant injuries in this segment of the study group.

Similarly, Gupta et al. conducted a prospective, observational study of 600 patients with blunt trauma who underwent whole-body CT and 101 who underwent limited scanning respectively after evaluation by the senior most emergency physician and the treating trauma surgeon [13]. The authors note that one or both of these physicians was willing to omit 35% of the individual scans performed and a critical action only occurred for 3 of these patients. The results of this study provide a framework upon which clinicians may discuss their clinical impression versus the risk of missed injury with a patient for the purposes of shared decision-making. Moreover, addressing the need for CT imaging (or lack thereof) with the trauma team may reduce CT ordering since the consulting physicians have been shown to be the driving factor for CTs deemed unnecessary by emergency physicians [16].

At the same time, in order to adequately assess the delicate balance between imaging exposure vs. risk of injury for patients, physicians need to have as complete as possible picture of a patient’s prior imaging history. Griffey et al. surveyed 155 emergency physicians to characterize their attitudes and practices with respect to CT utilization [17]. The investigators reported that 87% of the physician respondents indicated that they would utilize information regarding prior imaging history as a component of their discussions with patients regarding imaging options. We believe our report provides clinicians with some context of the prior imaging exposure for a cohort that represents the subset of trauma patients most likely to benefit from such shared decision making discussions.

## Limitations and future directions

Our study has several limitations that warrant discussion. Undoubtedly, our results underestimate the actual number of patients who had prior imaging and those who had more than one prior CT. We were able to review previous imaging records only within our hospital system, from 2009 to present, so CTs performed before that time would not be included. Further, we cannot account for scans performed in non-system facilities.

In addition, the retrospective design of our study allows for the possibility that bias may have been introduced both with respect to patients included, how they were managed, and their ultimate disposition. We do believe it is likely that all eligible patients were captured by our methods as we reviewed an inclusive electronic radiological database for all whole-body CTs ordered during the study period. Patients were only eligible for inclusion if at the discretion of their treating emergency physician a whole body CT was ordered and, ultimately, the patient was deemed safe for discharge directly from the ED. We are anecdotally not aware of physicians within our department specifically using standardized algorithms for selective CT in the setting of trauma. However, practice patterns for imaging as well as disposition surely vary between practitioners and, thus, this likely introduced some heterogeneity in the study group compared with the structure that a prospective protocol for imaging would alternatively provide.

Another limitation to our retrospective design is the possibility of bias and/or error in the data abstraction from the EMR and radiological databases. We did not perform an assessment of interobserver agreement for a sample of charts to assess the reliability of the chart review method. However, in view of the fact that the majority of data collected for the purpose of this study were objective (e.g. presence and type of prior CTs in the hospital system are provided in a vertical list by dates), we are confident that errors in the structured abstraction were likely minimal.

The characteristics of our study group may also raise concerns for external validity. Our local population is largely indigent and predominately Hispanic. It is unclear if patients from different socioeconomic and ethnic groups would be more or less at risk to have had prior imaging. Another source of potential concern for the generalizability of our results is with respect to our definition of low risk patients. We defined low risk patients as those discharged after ED evaluation. Future studies could improve upon our methods by utilizing low risk criteria identified previously [13].

## Conclusions

We found a positive history for prior CT for 25% of trauma patients who received whole-body CT scan but were discharged from the ED to home. Our results should encourage clinicians to evaluate prior CT imaging history and consider shared decision making/use of selective CT algorithms in case for which pretest assessment for risk of serious injury is relatively low.

## Abbreviations

CT: Cat Scan
ED: Emergency Department
EMR: Electronic Medical Record
